# Evolution of Functional Genomic Diversity During a Bottleneck

**DOI:** 10.1093/gbe/evaf107

**Published:** 2025-06-05

**Authors:** Flávia Schlichta, Stephan Peischl, Laurent Excoffier

**Affiliations:** Computational and Molecular Population Genetics (CMPG), Institute of Ecology and Evolution (IEE), University of Bern, Bern 3012, Switzerland; Swiss Institute of Bioinformatics, Lausanne 1015, Switzerland; Swiss Institute of Bioinformatics, Lausanne 1015, Switzerland; Interfaculty Bioinformatics Unit, University of Bern, Bern 3012, Switzerland; Computational and Molecular Population Genetics (CMPG), Institute of Ecology and Evolution (IEE), University of Bern, Bern 3012, Switzerland; Swiss Institute of Bioinformatics, Lausanne 1015, Switzerland

**Keywords:** background selection, associative overdominance, dominance coefficient, range expansion, pseudo-overdominance

## Abstract

Most species have been through population bottlenecks and range expansions, and the impact of these events on patterns of diversity has been well studied. In particular, it has been shown that initially rare neutral variants could readily fix on the front of range expansions or during bottlenecks, giving genomic signatures looking like selective sweeps. Here we expand on previous work by considering the dynamics of genomic diversity in (functional) regions harboring deleterious variants during bottlenecks or during range expansions modeled as serial founder effects. We find that regions with very low levels of diversity (troughs) looking like selective sweeps can also readily form in these functional regions. Additionally, their properties depend on the dominance level of deleterious mutations. The number of troughs is larger and increases more rapidly in regions with co-dominant deleterious mutations than in regions with recessive mutations. Interestingly, we find that genetic diversity declines less rapidly in regions with partially recessive mutations than in regions with codominant ones or in regions with only neutral mutations. These features are generally enhanced in low recombination regions and for intermediate selection coefficients. If most deleterious mutations in a genome are partially recessive, it follows that functional low recombination regions should better preserve genetic diversity during range expansions than neutral regions of the genome.

**Significance**

Most research on the effects of selection on genetic diversity has been done in equilibrium populations, leaving a gap in our understanding of how selection acting on functional regions interacts with demographic changes. Our study reveals strikingly different impacts of purifying selection on genetic diversity depending on the dominance coefficient of deleterious mutations. While codominant variants create diversity dips resembling selective sweeps, recessive mutations can preserve genetic diversity during bottlenecks through the relatively understudied phenomenon of pseudo-overdominance. Our findings show how selective and demographic forces interact in more complex ways than previously thought and emphasize the importance of further exploring these dynamics to accurately interpret genetic variation in natural populations.

## Introduction

Genetic variation is a product of the introduction of variants through mutation and the maintenance or removal of these variants by genetic drift or natural selection. Generally, selection is the driving force in large populations while drift becomes more important in smaller populations. Thus, to understand current levels of diversity, we must be able to disentangle these two competing forces. This is not an easy feat, however, since demographic changes can affect genomic diversity in ways similar to selection. For example, one of the earliest known signatures of positive selection is a decrease in heterozygosity around selected loci (Smith and Haigh 1974; Fay and Wu 2005), but such localized depletions of diversity are also a common outcome in populations having gone through bottlenecks (Jensen et al. 2005; Moinet et al. 2022) or after range expansions (Schlichta et al. 2022). Moreover, population expansions generate a coalescent tree similar to those from selective sweeps, creating a skewed site frequency spectrum (SFS; see e.g. [Tajima 1989; Slatkin and Hudson 1991; Wakeley 2009]). Akin yet distinct, the slow purging of deleterious mutations (or background selection, BGS) distorts the unfolded SFS in similar ways by producing an excess of low frequency derived variants (Desai et al. 2012; Cvijović et al. 2018). Contrastingly, an excess of high frequency variants can be caused by both recent selective sweeps and bottlenecks (Przeworski 2002; Zeng et al. 2007; Wakeley 2021; Moinet et al. 2022). The effect of positive or negative selection on nearby diversity is also dependent on local levels of recombination, with more pronounced effects in regions of low recombination (Begun and Aquadro 1992; Charlesworth et al. 1993; Hudson and Kaplan 1995; Stephan 2010; Pouyet et al. 2018).

Due to all these confounding characteristics, much of the early work on selection has been done assuming stable populations at mutation-drift-equilibrium, but several researchers have pushed for the development of more complex “null-models” (Cutter and Payseur 2013; Bank et al. 2014; Kern and Hahn 2018; Johri et al. 2020; Smith and Hahn 2024), and trying to account for BGS in selection scans (Huber et al. 2016). Recently, researchers have also started to study the combined effects of BGS and changes in population size to obtain better expectations of their consequences but also to improve past demography inference (Cvijović et al. 2018; Torres et al. 2020; Johri et al. 2021; Barroso and Ragsdale 2025). However, most studies investigating the action of selection are based on codominant variants (associated with dominance coefficient *h* = 0.5), which is not necessarily realistic (Di and Lohmueller 2024), since the fate of codominant and (partially) recessive selected variants can be quite different after demographic changes. For instance, after a bottleneck, deleterious recessive mutations are more easily purged, while codominant variants might actually increase in frequency (Kirkpatrick and Jarne 2000; Balick et al. 2015). Similarly, beneficial recessive variants spend longer times at low frequencies compared with dominant variants, thus having more opportunities to recombine into different backgrounds before reaching fixation, thus preserving more linked diversity during sweeps (Teshima and Przeworski 2006). Therefore, studies investigating the interaction between demographic changes and selection not only need to take into account the selection coefficients of mutations but also their levels of dominance.

Considering the importance of selection in general and of BGS in particular for shaping patterns of genomic diversity (McVean and Charlesworth 2000; Barton and Etheridge 2004; Stephan 2010; Charlesworth 2013; Pouyet et al. 2018; Gilbert et al. 2020) we aim here at expanding our previous work on the effect of range expansions on genomic diversity in neutral regions where we showed that regions of low diversity (troughs) would readily form during such expansions (Schlichta et al. 2022), and that their properties were resembling those of selective sweep (Moinet et al. 2022). In particular, we want to examine whether false selective sweep signals identified as troughs can still occur in functional regions after a series of bottlenecks, whether their dynamics are hampered or facilitated by BGS in regions harboring a mixture of neutral and deleterious variants with contrasting dominance coefficients, and to what extent recombination can modulate these signals.

## Results

### A Simple Bottleneck Mimics the Effect of a Range Expansion

In a previous paper (Schlichta et al. 2022), we studied the effect of a range expansion on patterns of genomic diversity. In particular, we investigated how the increase in frequency (and even fixation) of small chromosomal segments due to genetic surfing (Edmonds et al. 2004; Klopfstein et al. 2006; Hallatschek and Nelson 2008) generates patterns of low diversity on the front of a range expansion. Since range expansions can be modeled as a series of recurrent founder effects in a single population (DeGiorgio et al. 2011; Slatkin and Excoffier 2012), we compared patterns of diversity occurring during a bottleneck and during a range expansion in neutral (supplementary fig. S1, Supplementary Material online) and in functional (supplementary fig. S2, Supplementary Material online) regions. We found that conditional on the amount of diversity lost since the beginning of the bottleneck or the range expansion, bottlenecks of different sizes lead to virtually the same dynamics of trough formation than that observed during a range expansion. This observation justifies the use of simple bottleneck simulations in the following analyses rather than more complex spatially explicit range expansions and suggests that all results obtained from bottleneck simulations in the following should also apply to spatial range expansions.

**Fig. 1.**
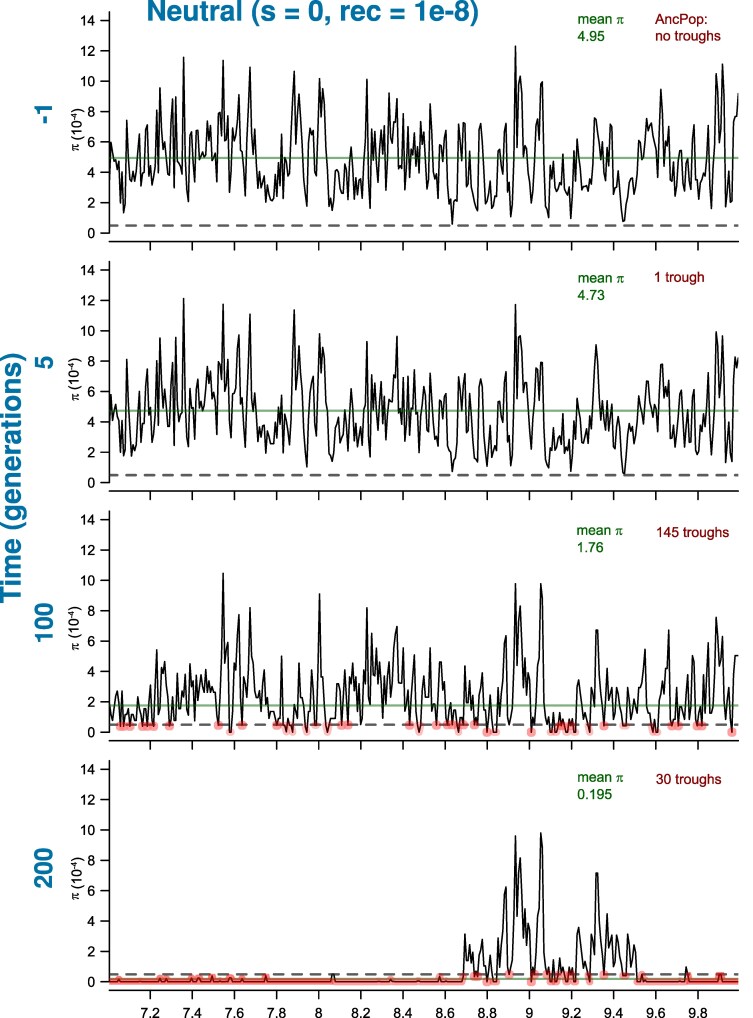
Genome scans of nucleotide diversity (π) during a bottleneck, for a neutral region of 3 Mb, highlighting the changes in the genomic landscape over time at the expansion edge. Four-time points are shown: −1, 5, 100, and 200 generations from the start of the bottleneck. Thus, generation −1 shows the ancestral population immediately before the bottleneck. *Y*-axis shows nucleotide diversity (π), and *X*-axis shows position in the genome. The horizontal dotted line indicates the threshold used to define troughs (10% of average ancestral diversity). The horizontal solid green line shows the average π value of the chromosome segment at each of the four-time points, and the exact value is shown in green on the top of each pane. Troughs are highlighted in red, with their total numbers (for the 10 Mb chromosome) shown in red on the top right of each pane (thus trough density is determined by dividing the total number of troughs by the chromosome length). Details on the parameters used in the neutral forward simulations can be found in the Materials and Methods section.

Thus, we performed forward simulations of a large ancestral population ($N_{\mathrm{anc}}=10,000$ diploid individuals) going through a sudden and severe bottleneck (*N*_Bot_ = 50). To track the decay of diversity over time, we sampled the bottlenecked population at 5-generation intervals and generated genomic diversity scans to characterize chromosome segments of highly reduced diversity, hereafter referred as troughs. Troughs were defined as regions with 10% or less of the average diversity observed in the ancestral population. An illustration of the genomic scans showing the global changes in patterns of genomic diversity and of troughs formation over time is shown in Fig. 1. Our simulation conditions were broadly based on human genomic diversity, see Materials and Methods for further details.

### Importance of Ancestral Population Size in Shaping Diversity Changes During a Bottleneck

**Fig. 2.**
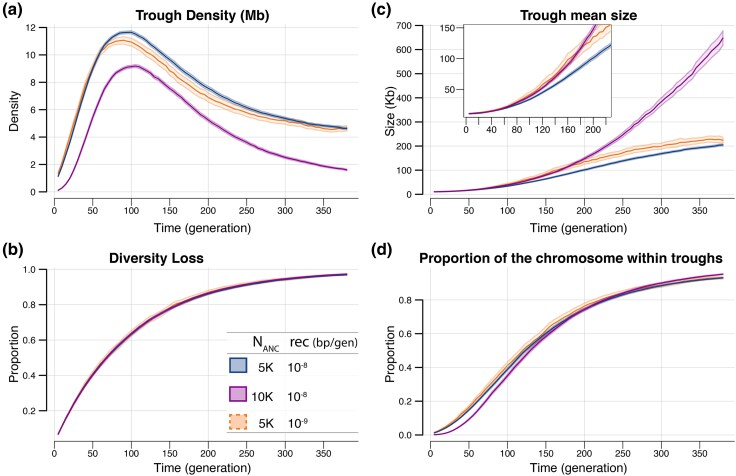
Statistics of trough dynamics during a bottleneck for neutral simulations in populations having different ancestral sizes ($N_{\mathrm{anc}}$) or different recombination rates (_*ρ*_ ). a) Trough density (number of troughs per Mb), b) diversity loss relative to the ancestral population, c) average trough size and d) proportion of the genome within troughs. Populations with smaller ancestral diversity (blue and orange lines) show higher trough density and smaller troughs compared with the population with larger ancestral sizes (magenta) before the onset of the bottleneck. Lower recombination (orange) only has a small effect in trough size and trough density. Importantly, despite different trough statistics and starting levels of diversity, all populations lose diversity at the same rate and a similar pattern is observed for the proportion of the genome within troughs, albeit with slightly different proportions between populations of different ancestral sizes. Shaded areas show 95% CI obtained from the bootstrap distribution of 10,000 bootstrap samples corresponding to resampling 100 genomic simulations with replacement.

Since the effects of BGS on genomic diversity can be approximated as a local reduction in effective population size (Charlesworth et al. 1993; Nordborg et al. 1996; Charlesworth 2013), we compare trough formation during a bottleneck in populations with varying ancestral sizes. Despite differences in $N_{\mathrm{anc}}$, trough formation during a bottleneck of a given size (*N*_Bot_) leads to qualitatively very similar dynamic: following population size reduction, the trough density (number of troughs per Mb) increases rapidly, reaches a maximum density at ∼ 2*N*_Bot_ generations, and then decreases as troughs begin to merge, resulting in an increase in their average size (Fig. 2). Quantitatively, however, the initial level of diversity does matter for trough dynamics. Indeed, we see that more troughs (Fig. 2a) of smaller size (Fig. 2c) are created when a population with a smaller ancestral size (with less standing genetic variability) goes through the same bottleneck as an initially larger population. However, the rate of diversity loss does not depend on the initial level of diversity as overall genetic diversity (average heterozygosity) diminishes identically when starting from a high or a low level of diversity (Fig. 2b). For populations with different ancestral size, the proportion of the genome within troughs during the bottleneck (Fig. 2d) is found to be only slightly different in the first ∼ 2*N*_Bot_ generations, and then these populations evolve remarkably similarly, with an increase akin to that observed for relative diversity loss. Due to this similarity, in the following sections we will refrain from showing the proportion of the chromosome within troughs and focus on relative diversity loss instead. In addition, we see that recombination rate slightly affects trough formation, with regions of low recombination showing comparatively larger but less numerous troughs than regions with higher recombination rates (Fig. 2a and c). But again, the overall change in diversity loss does not depend on recombination rate (Fig. 2b).

### Effect of Deleterious Mutations in the Ancestral Population

We then simulated realistic BGS in the ancestral population by adding deleterious mutations at rate $\mu_{\mathrm{Del}}=\;1.1\times 10^{-9}$ in a portion of the genome with lower recombination rate $\rho_{\mathrm{Del}}=5\times 10^{-9}$, since regions with recombination rate in humans higher than 1cM/Mb (1 × 10^−8^) are largely unaffected by BGS (Pouyet et al. 2018). Deleterious variants had a fixed selection coefficient *s* and identical dominance coefficient *h* for all variants (either highly recessive with *h* = 0.1, or codominant *h* = 0.5). We compare the diversity of BGS-affected regions to neutral regions in the ancestral population using a common measure of BGS strength, *B*, which is defined as the ratio $\pi_{\mathrm{BGS}}/\pi_{\mathrm{Neutral}}$, where *π* represents the average diversity in a given region. Thus, the *B* metric directly compares levels of diversity between neutral and selected regions of the genome in the ancestral population. As expected, the presence of deleterious mutations over a genomic region affects the level diversity present before the bottleneck, which is shown in Table 1 for various combinations of selection coefficients, dominance levels and recombination rates. *B* values lower than one indicate lower levels of diversity in functional regions, whereas *B* values larger than one indicate higher levels of diversity in functional regions. For co-dominant deleterious mutations, the ancestral level of diversity in the selected chromosome is always lower than in the neutral chromosome, with much lower diversity with higher selection coefficients and in regions of low recombination, in line with BGS expectations (Charlesworth et al. 1993; Hudson and Kaplan 1995). Contrastingly, in the presence of partially recessive variants, the level of diversity can be either lower or higher than that in the neutral chromosome. For the same recombination rate, diversity goes down with increasing selection coefficients. However, in regions of low recombination, nucleotide diversity can be much higher in the selected than in the neutral chromosome, especially in the presence of deleterious mutations with small selection coefficient (*s* = 0.0001). This result can be attributed to the presence of associative (pseudo-)overdominance (APOD), where an apparent heterosis in neutral loci can arise due to close linkage to overdominant loci (Sved 1968; Ohta and Kimura 1969) or, as it happens in this particular case, due to the masking of several recessive deleterious alleles in strong linkage disequilibrium (Ohta 1971; Zhao and Charlesworth 2016; Gilbert et al. 2020; Waller 2021; Glémin 2022; Abu-Awad and Waller 2023). APOD has been already observed in other simulations studies (Smith and Hahn 2024) and in real organisms (Schou et al. 2017). This transition from BGS to APOD is thus expected in regions of low recombination where recessive mutations at multiple loci are captured in repulsion on different haplotypes (Gilbert et al. 2020).

**Table 1:** BGS intensity (measured by *B* values) for cases with different selection intensities (*s*), dominance coefficients (*h*) and recombination rates

Dominance level *h*	Recombination rate^a^	*s*	Nucleotide diversity in BGS regions ($\pi_{\mathrm{BGS}}$)	*B*
NA^b^	Intermediate	0.0000	0.00053	1
0.1	Intermediate	0.0001	0.00059	1.11
		0.0010	0.00042	0.79
		0.0015	0.00039	0.75
	Low	0.0001	0.00090	1.70
		0.0010	0.00059	1.11
		0.0015	0.00047	0.90
0.5	Intermediate	0.0001	0.00047	0.88
		0.0010	0.00034	0.64
		0.0015	0.00033	0.62
	Low	0.0001	0.00040	0.75
		0.0010	0.00017	0.36
		0.0015	0.00014	0.27

^a^Intermediate: $5\times 10^{-9}$ per bp per generation. Low: $1\times 10^{-9}$ per bp per generation.

^b^Simulations with *s* = 0 are neutral, and thus dominance coefficient is not relevant here.

### Effect of Deleterious Mutations During the Bottleneck

**Fig. 3.**
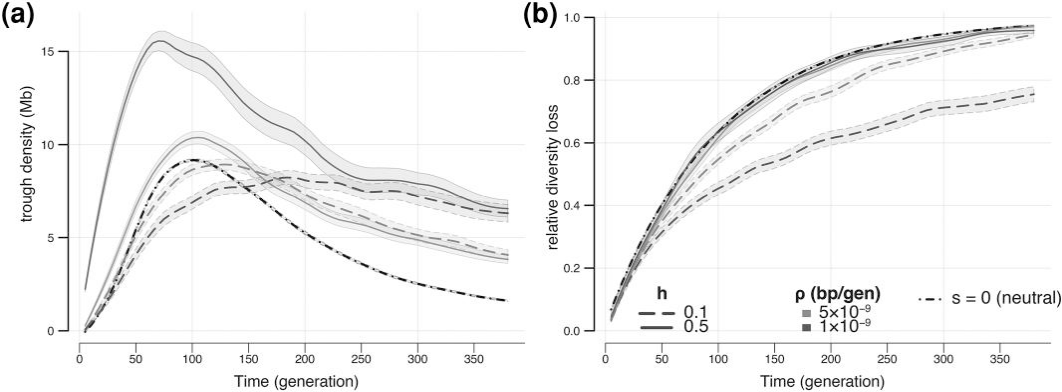
Effect of recombination rate (*ρ*) and dominance levels (*h*) on trough density a) and diversity loss b) during a bottleneck. The selection coefficient of deleterious mutation was set to *s* = 0.0015 for both recessive (*h* = 0.1, dashed lines) and codominant (*h* = 0.5, solid lines) variants. Results for chromosomes with low recombination ($\rho =1\times 10^{-9}$ per bp per generation) are shown in purple, whereas those for regions of intermediate recombination ($\rho =5\times 10^{-9}$ per bp per generation) are shown in orange. Note that the recombination rate for the neutral case is $\rho \;=10^{-8}$ per bp per generation. Shaded areas show 95% CI obtained from 10,000 bootstrap iterations. Note that the neutral model (*s* = 0) is represented in a black dash-dotted line and was carried out in larger chromosomes (100 Mb instead of 20 Mb for selected chromosomes), leading to smaller confidence intervals, but with similar average statistics (see supplementary fig. S4, Supplementary Material online).

**Fig. 4.**
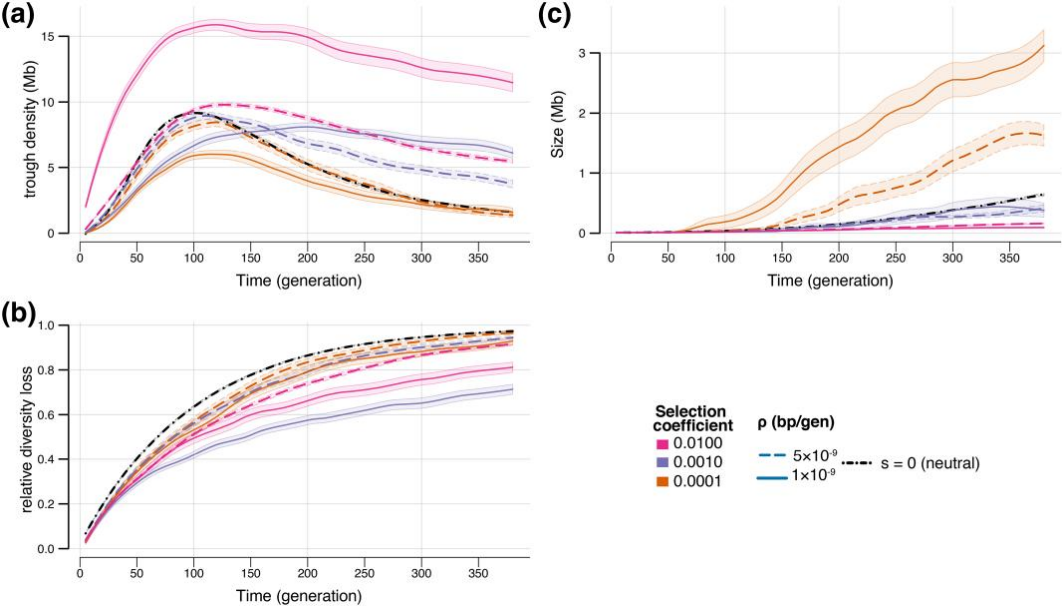
Effect of selection intensity (*s*) on trough density a), diversity loss b) and trough size c) during a bottleneck. All cases shown are for simulations with partially recessive deleterious mutations (*h* = 0.1). Results for chromosomes with a low recombination rate ($\rho =10^{-9}$ per bp per generation) are shown with a solid line, whereas those with an intermediate recombination rate ($\rho =5\times 10^{-9}$ per bp per generation) are shown with a dashed line. Note that the recombination rate for the neutral case is $\rho =10^{-8}$ per bp per generation. Orange lines represent results obtained for chromosomes including deleterious mutation of very small effect (*s* = −0.0001), whereas those in purple are for chromosomes with mutations 10× stronger (*s* = −0.001), and those in pink with mutations 100× stronger (*s* = −0.0100). Shaded areas show 95% CI obtained from 10,000 bootstrap iterations. Note that the neutral model (*s* = 0) is represented in a black dash-dotted line and was carried out in larger chromosomes (100 Mb instead of 20 Mb for selected chromosomes), leading to smaller confidence intervals, but with similar average statistics (see supplementary fig. S4, Supplementary Material online).

Whereas we have seen that levels of (neutral) ancestral diversity have a mild effect on trough properties during a bottleneck (Fig. 2a and b), the rate at which diversity is lost is not affected by ancestral diversity (Fig. 2c). Contrastingly, we find that in the presence of deleterious mutations, both trough dynamics and rate of diversity loss differ from what is expected under neutrality. In Fig. 3 we show the effect of dominance level and recombination rate on genomic diversity, whereas in Fig. 4 we show the effect of selection intensity and recombination for highly recessive mutations.

#### Chromosomes With Recessive Deleterious Mutations Preserve Diversity Better

In the presence of co-dominant deleterious mutations, trough density on the selected chromosome is always higher than on the neutral chromosome (Fig. 3a), but the rate of diversity loss is identical for the selected and the neutral chromosome (Fig. 3b). Therefore, the behavior of a chromosome with co-dominant mutations is essentially following what is expected from a mere reduction in effective population size by BGS. Contrastingly, compared with a neutral chromosome, one with recessive deleterious mutations shows a delay in trough formation (Fig. 3a), and importantly, a less rapid loss of diversity during the bottleneck (Fig. 3b). In other words, initial diversity is better preserved during a bottleneck in regions harboring recessive mutations than in neutral regions or than in regions including co-dominant mutations, and this is especially true in regions of low recombination (Fig. 3b). This slower loss of diversity in presence of recessive mutations is illustrated by diversity genome scans done at different time points in supplementary fig. S3, Supplementary Material online. This result suggests that pseudo-overdominance can also develop during bottlenecks and not only in the ancestral population. This is surprising since one would have expected drift to be stronger than selection during these periods of very small sizes. However, we see that the difference in diversity loss does not immediately emerge, it only appears after 30 to 50 generations of bottleneck (i.e. after 0.6 *N*_Bot_ or 1 *N*_Bot_ generations, respectively). Note that chromosomes with dominant deleterious mutations (*h* = 0.8) tend to have a slightly faster rate of diversity loss than those with recessive, codominant, or neutral mutations (see supplementary fig. S5, Supplementary Material online).

#### Importance of Strength of Selection in Chromosomes With Recessive Variants

Since patterns of diversity in chromosomes with co-dominant deleterious mutations can be simply explained by a change in ancestral effective population size, we then focused on chromosomes harboring recessive mutations associated with different selection coefficients (Fig. 4). Chromosomes with intermediate recombination rate (*r* = 5 × 10^−9^ per bp per generation) harboring slightly deleterious mutations (*s* = −0.0001) show slightly fewer troughs per Mb than neutral chromosomes (Fig. 4a), but these troughs are larger (Fig. 4c). Similarly, mutations occurring on chromosomes with lower recombination rates (*r* = 1 × 10^−9^ per bp per generation) show much fewer but much larger troughs (Fig. 4a and c).

Chromosomes with deleterious mutations having a 10 times larger selective disadvantage (*s* = −0.001) and intermediate recombination rate, show a different pattern. Trough sizes are very similar to those of neutral chromosomes during the bottleneck (Fig. 4c), but trough density is initially lower than that of neutral chromosomes but then remains relatively high in the later stages of the bottleneck (Fig. 4a). As is visible in the right pane of supplementary fig. S3, Supplementary Material online in generation 200, many troughs can be seen interrupted by very small peaks (islands) of diversity slightly overshooting the trough definition threshold, a pattern not seen at the same time for neutral chromosomes (left pane of supplementary fig. S3, Supplementary Material online), explaining the difference in trough density between these cases. Importantly, and as already seen in Fig. 3b, the rate of diversity loss is much slower in low recombination chromosomes (Fig. 4b), so that low recombination regions harboring recessive variants should better preserve diversity during bottlenecks.

However, the study of low recombination chromosomes (*r* = 10^−9^ per bp per gen) including recessive mutations (*h* = 0.1) with even larger selection disadvantage (*s* = −0.01, Fig. 4) reveals that, contrary to what happens in chromosomes with intermediate recombination rates (*r* = 5 × 10^−9^ per bp per gen), the rate of diversity loss is not inversely correlated with selective disadvantage, as these chromosomes lose diversity more rapidly than chromosomes harboring mutations with intermediate s (−0.001). It therefore seems that in low recombination regions, the presence of mutations with intermediate selection coefficients (*s* = −0.001) is more efficient in preventing diversity loss than when there are only mutations with either much smaller (*s* = −0.0001) or much larger (*s* = −0.01) effect (Fig. 4b). To further explore the limits of these effects, we also simulated recessive deleterious variants with even more extreme selection coefficients (0.25 and 0.00001, supplementary fig. S6, Supplementary Material online), which are shown to have a minimal impact on relative diversity loss. Note that for chromosomes with codominant variants, the strength of selection has no effect on the rate of diversity loss during bottlenecks, and it has minimal impact on trough density and trough sizes (supplementary fig. S7, Supplementary Material online). In summary, and in line with BGS dynamics (Nordborg et al. 1996; Good et al. 2014; Barroso and Ragsdale 2025) intermediate selection coefficients paired with low recombination have the largest impact on diversity loss.

### Genomic Distribution of Deleterious Mutations

**Fig. 5.**
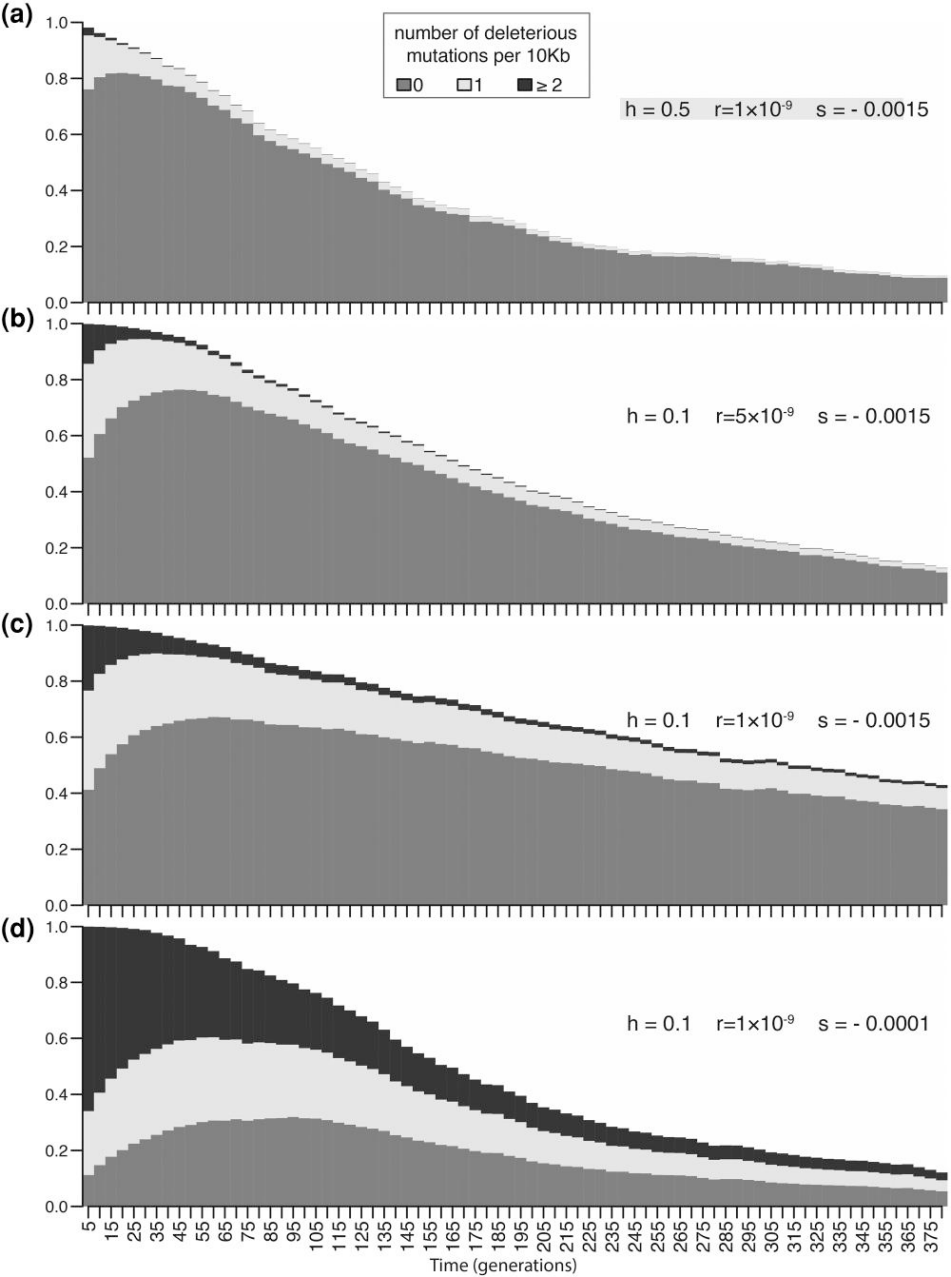
Evolution of the proportion of the genome in islands of diversity (colored bars) including 0, 1, or ≥2 recessive mutations per 10 Kb window. a) chromosomes of low recombination rate including codominant mutations with selective disadvantage *s* = −0.0015. (b–d) chromosomes with highly recessive mutations (*h* = 0.1). b) *s* = −0.0015 and intermediate recombination (*ρ* = 5×10^−9^ per bp per generation); c) same *s* and *h* as b) but in regions of low recombination (*ρ* = 10^−9^ per bp per generation); d) same *h* and recombination as c) but with *s* = −0.0001.

To better understand what is driving the slower loss of diversity in chromosomes with recessive deleterious mutations, we have examined how these deleterious mutations were distributed across regions of low (troughs) or high (islands) diversity and how this would change over time. The evolution of the mutation load in islands of diversity during bottlenecks is visualized in Fig. 5. For codominant markers (Fig. 5a) the genome is invaded by troughs and most genomic windows within islands of diversity harbor no deleterious mutations. In chromosomes with intermediate selected (recessive) mutations and recombination rate (Fig. 5b), the speed at which troughs invade the genome is slower, and islands of diversity can sometimes maintain 1 deleterious mutation per 10 Kb during the whole bottleneck and very few windows of 10 Kb show 2 or more deleterious mutations. In regions of low recombination with recessive mutations, there is a much higher proportion of the genome in islands of diversity during the bottleneck (Fig. 5c), explaining the lower rate of diversity loss for this parameter combination (Figs. 3b and 4b). Note that we also see here a higher proportion of segments of 10 Kb in islands that harbor at least 1 deleterious mutation (Fig. 5c), and because these islands are relatively large (e.g. around 4 Mb at the onset of the bottleneck and 200 and 120 Kb after 50 and 100 generations, respectively; see supplementary fig. S8, Supplementary Material online), each island contains several sites with deleterious mutations, allowing for the emergence of pseudo-overdominance. With very weakly selected recessive mutations (*s* = −0.0001), a much larger proportion of the islands of diversity shows 2 or more deleterious mutations (Fig. 5d), but there are proportionally fewer islands of diversity than for more deleterious recessive mutations (*s* = −0.0015, as in Fig. 5c). While the evolution of the proportion of the genome in troughs matches the evolution of diversity loss observed in Figs. 3b and 4b, we see that the fewer islands of diversity observed in chromosomes with very weakly deleterious recessive mutations show a large excess of multiple deleterious mutations (Fig. 5d), which is compatible with the emergence of pseudo-overdominance (Glémin 2022; Abu-Awad and Waller 2023).

## Discussion

The effect of bottlenecks or range expansions on functional genomic diversity can vary substantially from what is expected in neutral regions as described in (Schlichta et al. 2022). Indeed, we find that trough formation is more rapid in regions harboring co-dominant deleterious mutations than in neutral regions or in regions harboring recessive mutations (Fig. 3). Contrastingly, the formation of troughs is slowed down in functional regions with recessive deleterious variants as compared to neutral regions, resulting in a comparatively slower loss of diversity during the bottleneck (Fig. 3). The preservation of genetic diversity in the presence of deleterious variants can only be explained by the emergence of some form of associative overdominance (AOD) (Sved 1968; Ohta and Kimura 1969) and probably of associated pseudo-overdominance (Glémin 2022; Abu-Awad and Waller 2023), where several deleterious alleles are maintained in repulsion on different haplotypes. Whereas AOD has been shown to occur recently in currently large populations (Leitwein et al. 2019; Gilbert et al. 2020), its occurrence in bottlenecked populations was unexpected, but it emerges only for certain combinations of intermediate and small selection coefficients and low recombination rates, the exact combination of which would deserve to be investigated theoretically. Nevertheless, because regions of low recombination are widespread in many species (Stapley et al. 2017) and intermediate selection coefficients of the magnitude used here (e.g. *s* = ∼0.001) have been inferred in several species (Gallet et al. 2012; Huber et al. 2017; Kim et al. 2017; Vaughn and Nielsen 2024), conditions for the appearance of AOD during expansions may not be uncommon. The fact that diversity preservation is more pronounced in regions of low recombination suggests that the linkage of multiple low effect mutations in the same chromosomal regions allows selection to be still operational during a bottleneck when the population size is extremely low (*N*_Bot_ = 50). This goes against the belief that selection only operates at a given locus if *Ns* is larger than 1 (*Ns* ranged from 0.005 to 0.75 in our simulations) (Kimura 1962), but it is possible in the case of a bottleneck because selection acts at the same time on a large number of selected variants that were created in the ancestral population. Obviously, if the bottleneck were to last for a long time, one would indeed expect the population to reach a new equilibrium, where the fate of new mutations would indeed depend on their associated *Ns* value.

Whereas it had been previously shown that BGS could lead to regions of low diversity (troughs) mimicking selective sweeps in stationary populations under very restricted conditions (Schrider 2020), our work shows that troughs can steadily arise during bottlenecks and that they are almost as likely to emerge in functional regions harboring negatively selected variants than in neutral regions, suggesting that signals looking like selective sweeps are also expected in these regions during bottlenecks or range expansions. In this respect, BGS does not act as a buffering mechanism preventing the emergence of these selective sweep looking regions. In the presence of mainly co-dominant deleterious mutations, one would even expect that these signals be more prevalent than for neutrally evolving regions (Fig. 3). However, even though most of the genome will harbor a mixture of dominant, co-dominant and (partially) recessive mutations, it is widely accepted that most functional mutations are partially recessive (Di and Lohmueller 2024), suggesting that results we obtained for recessive mutations should be seen more often, and that troughs will develop more slowly in functional regions than in neutral regions, especially if recombination rates are low. Trough dynamics and genome diversity evolution are also affected by mutation selection intensity (Fig. 4) and the exact pattern of diversity will depend in complex interactions between recombination rates, dominance levels and selective effects of mutations. Because functional regions with co-dominant mutations lose diversity at the same rate as neutral region, considering that BGS leads to a mere reduction in population size (Charlesworth et al. 1993; Hudson and Kaplan 1995) is a good approximation (Table 1, Fig. 3b, supplementary figs. S5 and S7, Supplementary Material online). Contrastingly, functional regions with mainly recessive deleterious mutations should lose genetic diversity more slowly than neutral regions, and this loss is slowest in low recombination regions harboring partially recessive mutations of intermediate effects (Fig. 4), results also previously found in Gilbert et al (2020). While codominant markers drawn from a more realistic gamma distribution of fitness effects (DFE) continue to have the rate of diversity loss close to those of neutral regions, trough density is only higher than neutrality if the average selection coefficient is sufficiently large (supplementary fig. S7, Supplementary Material online). Similarly, regions with recessive deleterious variants following a gamma distribution behave very close to neutrality (supplementary fig. S9, Supplementary Material online), since diversity is most affected by recessive variants of intermediate effect (Fig. 4), which are rare with L-shaped gamma distributed DFEs. However, we note that the gamma DFE of recessive variants can still produce effects comparable to a uniform DFE when recombination rate is low enough (supplementary fig. S9, Supplementary Material online).

While this work initially aimed at studying the trough formation in functional regions during a bottleneck or a range expansion, we believe that the observed difference in loss of diversity between functional and neutral regions is an interesting result. Indeed, the finding that genomic diversity is better preserved in low recombination regions harboring recessive variants is a testable hypothesis. One could indeed compare neutrally evolving regions with functional regions, since they are expected to show different rates of loss of diversity (Fig. 3) or compare low recombination with high recombination regions, the latter being virtually unaffected by BGS (Pouyet et al. 2018). However, this comparison might be difficult in practice, since these regions might have different initial levels of diversity (Table 1), and functional diversity might always remain lower than neutral diversity during the expansion. Therefore, one would need to have an estimation of the ancestral levels of diversity in those regions, before the start of the bottleneck or range expansion, to be able to make meaningful comparisons.

## Materials and Methods

### Forward Simulations

We used forward simulations to investigate the effects of recombination and selection on genomic diversity in a diploid population going through a bottleneck (as a surrogate for a population range expansion, see below). We performed all simulations with SLiM 3 (Haller and Messer 2019). First, we simulated the diversity of a stable ancestral population made up of $N_{\mathrm{anc}}$ diploid individuals ($N_{\mathrm{anc}}\;\mathrm{was}\;\mathrm{set}\;\mathrm{to}\;10,000\;\mathrm{unless}\;\mathrm{specified}\;\mathrm{otherwise})\;$ for $10\times N_{\mathrm{anc}}$ generations to reach mutation-drift-selection equilibrium. After this burn-in period, we simulated a major instantaneous bottleneck, reducing the population size to *N*_Bot_ = 50 individuals. The population remains at this small size during the rest of the simulation and evolves in isolation for 380 generations (or 7.6 *N*_Bot_ generations), an arbitrary time but usually sufficiently long to lose most ancestral diversity. We monitored genetic diversity change by sampling forty diploid genomes every five generations, totaling 76 time-samples for every simulated replicate. We then performed 100 simulation replicates per combination of demographic and selection scenarios.

### Neutral and Deleterious Mutation Rates

For simulations involving deleterious mutations, the genome of each individual was made up of two independent chromosomes of equal length, and neutral diversity was created by using the same neutral mutation rate $\mu_{\mathrm{Neu}}\;=1.25\times 10^{-8}$ per bp per generation on each chromosome. Note that the value of $\mu_{\mathrm{Neu}}\;$ is within the range of the average genome-wide rates estimated in humans (Kong et al. 2012; Narasimhan et al. 2017). BGS was simulated on only one of the two chromosomes, by implementing a deleterious mutation rate ($\mu_{\mathrm{Del}}$). We set the deleterious mutation rate to 1.1×10^−9^ based on the observed reduction of diversity in humans and the theoretical results of Hudson and Kaplan (Hudson and Kaplan 1995). More specifically, if the total recombination rate is large relative to the strength of selection against heterozygotes (sh), it is predicted that genetic diversity reduced by BGS ($\pi_{\mathrm{BGS}}$) is equal to $\pi_{\mathrm{Neu}}\exp\;(-\mu_{\mathrm{Del}}/\rho_{\mathrm{Del}})$ (Hudson and Kaplan 1995), where $\pi_{\mathrm{Neu}}$ is the diversity of neutral regions and $\rho_{\mathrm{Del}}$ is the per bp recombination rate in the chromosomal region harboring deleterious mutations. The ratio $B=\pi_{\mathrm{BGS}}/\pi_{\mathrm{Neu}}$ is a common measure of the effects of BGS and, when comparing diversity of human African populations in regions with high and low recombination rates taken as proxy for neutral and BGS regions, respectively (Pouyet et al. 2018), *B* is found to be approximately =0.8 (e.g. McVicker et al. 2009). From this observation and under the approximation of Hudson and Kaplan (1995), we infer that $\mu_{\mathrm{Del}}=-\log\;(B)\;\;\times \rho_{\mathrm{Del}}=1.1\times 10^{-9}$, with $\rho_{\mathrm{Del}}=5\times 10^{-9}$ (see below).

In order to test if we can approximate the effect of BGS by a mere reduction of effective population size (Charlesworth et al. 1993; Nordborg et al. 1996; Charlesworth 2013), we also ran simulations with only neutral mutations and investigated the effect of different ancestral population sizes (10,000 and 5,000 diploid individuals), leading to various levels of ancestral diversity. Note that these pure neutral simulations were done for 100 Mb genomes, while scenarios with selection were done on a smaller 20 Mb genome, for the sake of computational efficiency. We evaluated the effect of genome size on simulated statistics in a few cases (supplementary fig. S4, Supplementary Material online) and did not find any significant effect of simulated genome size on trough density and rate of diversity loss, so that all results including selection presented here have been computed from 20 Mb genomes.

### Properties of Deleterious Mutations

Deleterious mutations either had a single fixed selection coefficient, *s*, or selection coefficients were drawn from a DFE for each new mutation. For the DFE, we used a Gamma distribution of selection coefficient values around a given mean *s* and a shape parameter (*a* = 0.23) assumed to fit human functional diversity (Eyre-Walker et al. 2006; Harris and Nielsen 2016). Note that this DFE yields a majority of nearly neutral deleterious mutations and a long tail with higher selective values. The dominance coefficient (*h*) of deleterious mutations in heterozygote individuals was assumed identical for all mutations within replicates such that the fitness of heterozygotes is 1−hs, whereas the fitness of homozygotes is 1−s. We simulated either codominant mutations (*h* = 0.5) or partially recessive mutations (*h* = 0.1). The chosen value of *h* = 0.1 for recessive deleterious mutations is in the range of values previously estimated from humans (0.05 to 0.25; see i.e. Agrawal and Whitlock 2011; Di and Lohmueller 2024; Kyriazis and Lohmueller 2024). Individual fitness over the whole genome was then computed multiplicatively across loci for each individual. Finally, the recombination rate was uniform within each chromosome, but different for neutral and selected chromosomes. The recombination rate of the neutral chromosome was set to a “high” rate ($1\times 10^{-8}$ per bp per generation), whereas that of the selected chromosome was either set to an “intermediate” ($5\times 10^{-9}$) or “low” ($1\times 10^{-9}$) rate. These values follow a previous work (Pouyet et al. 2018) showing that the human genome can be safely classified in BGS-affected regions if its recombination rate is smaller than 1cM/Mb or 10^−8^ per bp per generation.

### Summary Statistics

Simulated genome sequences were output in VCF format, and then processed by VCFtools (Danecek et al. 2011), and various custom scripts were deployed in R 4.3 (R Core Team 2023). The genomes were scanned in 10 Kb overlapping windows and expected heterozygosity (H=2p(1−p), where *P* is the allele frequency) was calculated for each polymorphic locus, summed over all loci, and then divided by the window length, to get the expected nucleotide diversity (*π*) per bp. As before (Schlichta et al. 2022), we used a threshold of 10% of initial diversity (mean diversity of the ancestral population) to determine if genomic windows were within troughs or islands of diversity. Troughs were then defined as adjacent windows with diversity at or below this 10% threshold, while islands of diversity were regions with diversity above that threshold. Thus, the minimum size of a trough or of an island is one genomic window (10 Kb). Following a previous approach (Schlichta et al. 2022), we summarized the diversity landscape by calculating trough density (number of troughs per Mb), trough size (length), relative diversity loss (diversity lost compared with the ancestral diversity), and the proportion of the genome within troughs (i.e. under the established threshold). To obtain confidence intervals, for each of the 76 sampled time points, all statistics were bootstrapped. More specifically, for each generation we resampled 10,000 times with replacement from the 100 independent simulations, recomputing the trough statistics for each bootstrap sample. The 2.5% and 97.5% quantiles of the obtained distribution were then used to delimit a 95% confidence interval. In order to facilitate visualization, the results obtained with 20 Mb genomes were smoothed using a local polynomial regression implemented in R through the function “loess,” using a local fitting of 0.4 (argument “span”), unless stated otherwise.

## Supplementary Material

evaf107_Supplementary_Data

## Data Availability

Scripts used to run simulations and perform data analyses will be publicly available as a git repository (github.com/CMPG/BGS_w_BTNCK).
