# Supplementary material for: Evolution of Functional Genomic Diversity During a Bottleneck
Source: Genome Biol Evol. 2025 Jun 5;17(6):evaf107. doi: 10.1093/gbe/evaf107 (PMC12204323; doi:10.1093/gbe/evaf107)
Supplement: evaf107_Supplementary_Data [file evaf107_supplementary_data.docx]

**Evolution of functional genomic diversity during a bottleneck**

Supplementary Materials

Flávia Schlichta^1,2, *^, Stephan Peischl^2,3^ and Laurent Excoffier^1,2, *^

^1^ Computational and Molecular Population Genetics (CMPG), Institute of Ecology and Evolution (IEE), University of Bern, 3012 Bern, Switzerland

^2^ Swiss Institute of Bioinformatics, 1015 Lausanne, Switzerland

^3^ Interfaculty Bioinformatics Unit, University of Bern, 3012 Bern, Switzerland

* Corresponding authors: flavia.schlichta@unibe.ch; laurent.excoffier@unibe.ch

ORCID:

F. Schlichta: 0000-0002-8845-3623

S. Peischl: 0000-0002-0474-6104

L. Excoffier: 0000-0002-7507-6494

Emails:

F. Schlichta: flavia.schlichta@unibe.ch

S. Peischl: stephan.peischl@unibe.ch

L. Excoffier: laurent.excoffier@unibe.ch


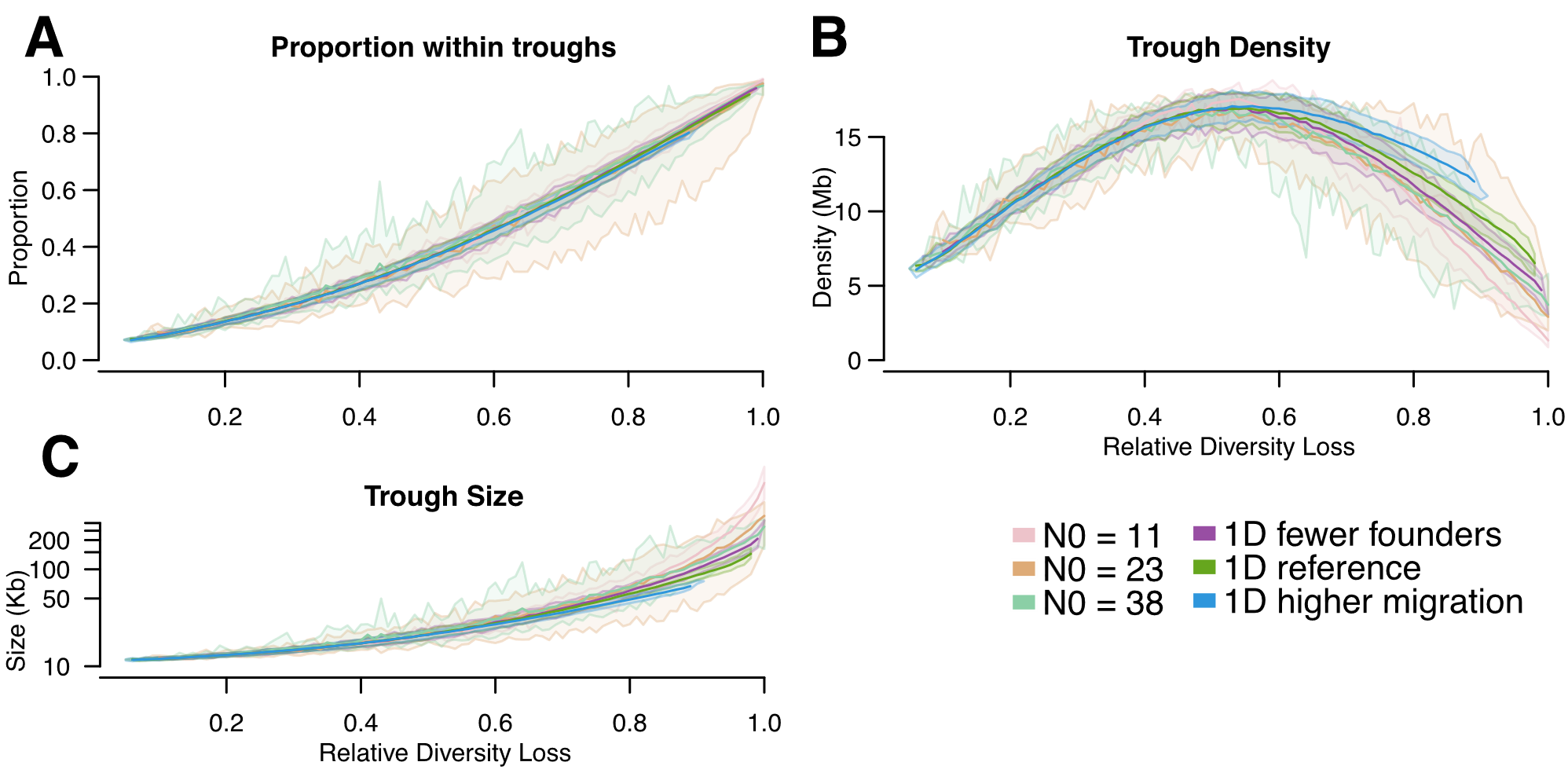


Figure S1: Trough formation dynamics during bottlenecks or range expansions starting from the same level of ancestral diversity. A: proportion of the genome in troughs (in regions of the genome with less than 10% of ancestral diversity). B: Number of troughs per Mb. C: Trough size. All trough statistics are plotted as a function of the proportion of diversity lost since the beginning of the bottleneck. We report here trough statistics for bottlenecks of different sizes (N=11, 23 or 38 diploids) and for one-dimensional (1D) range expansions with different properties, either with fewer founders colonizing a new habitat or with higher migration rates between nearby demes, as compared to a reference scenario, as described in Schlichta et al. (2022). Solid lines represent the mean statistic among all replicates and the shaded areas delineate 95% of the observed values.


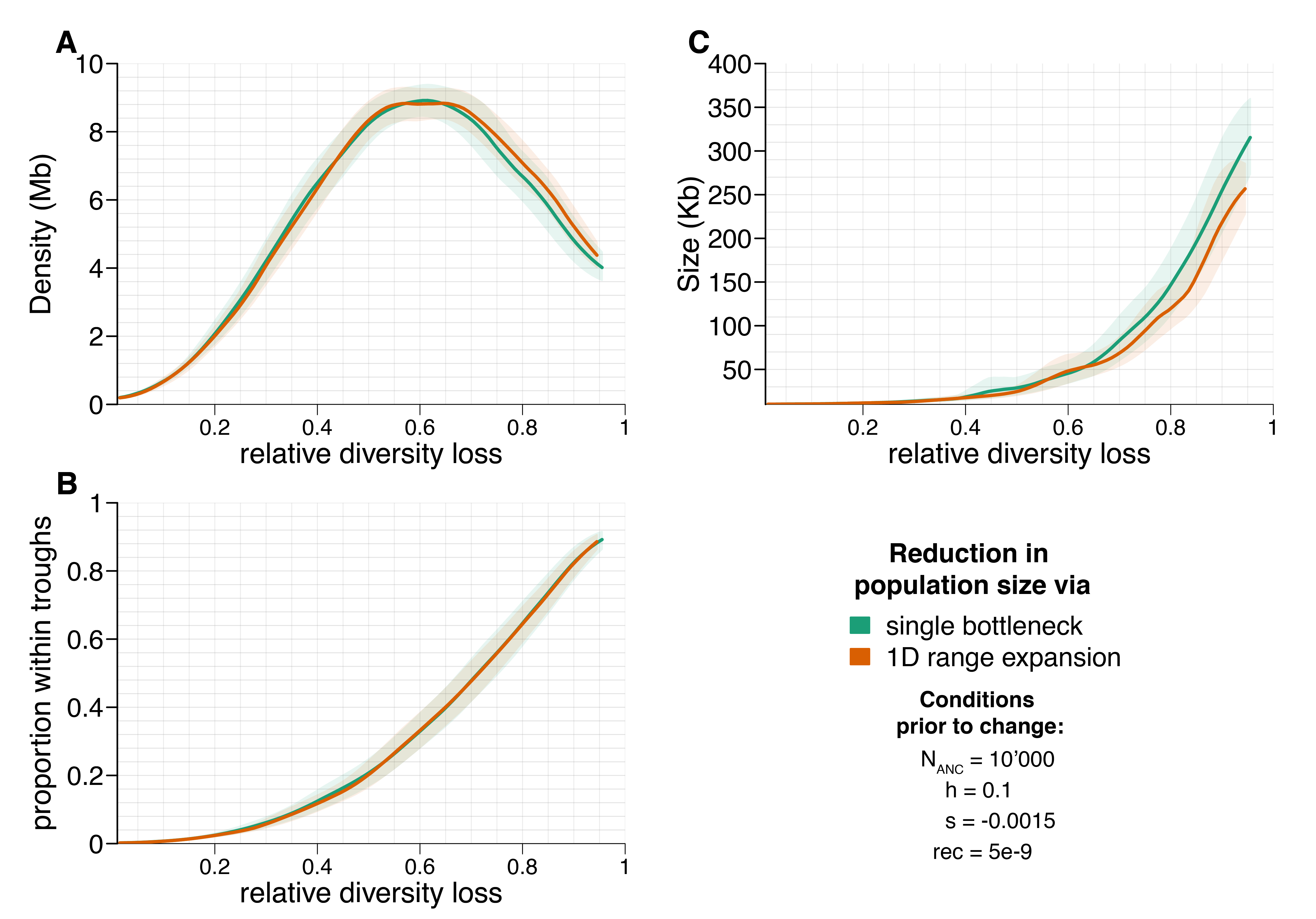


Figure S2: Trough density (A), proportion of the genome within troughs (B) and trough size (C) as a function of relative diversity loss in function regions, for two demographic scenarios: a single bottleneck (green) and a one-dimensional stepping-stone range expansion (orange). The lines represent only chromosomes under the influence of BGS and these populations have started from the same ancestral conditions: $N_{anc}$ = 10,000, highly recessive (h = 0.1) deleterious mutations (s = 0.0015) and with intermediate recombination rate (5e-9 per bp per geneneration). The bottleneck is simulated as described in the Materials and Methods section, whilst the 1D expansion occurs as follows: the first deme is created by a given number of founders N_founders_ coming from the ancestral population. This deme then grows exponentially to reach carrying capacity N_MAX_ within a given number of generations T_grow_, during which the populations exchange migrants at rate M. After that, this deme sends out N_founders_ individuals to form the newest edge deme, which will then proceed to grow to N_max_ within T_grow_ generations while exchanging migrants with adjacent demes. This process is repeated until 100 new demes are formed. Shaded areas show 95% CI obtained from 10,000 bootstrap iterations. The results were smoothed through a local polynomial regression (span = 0.4), see Material and Methods for details.


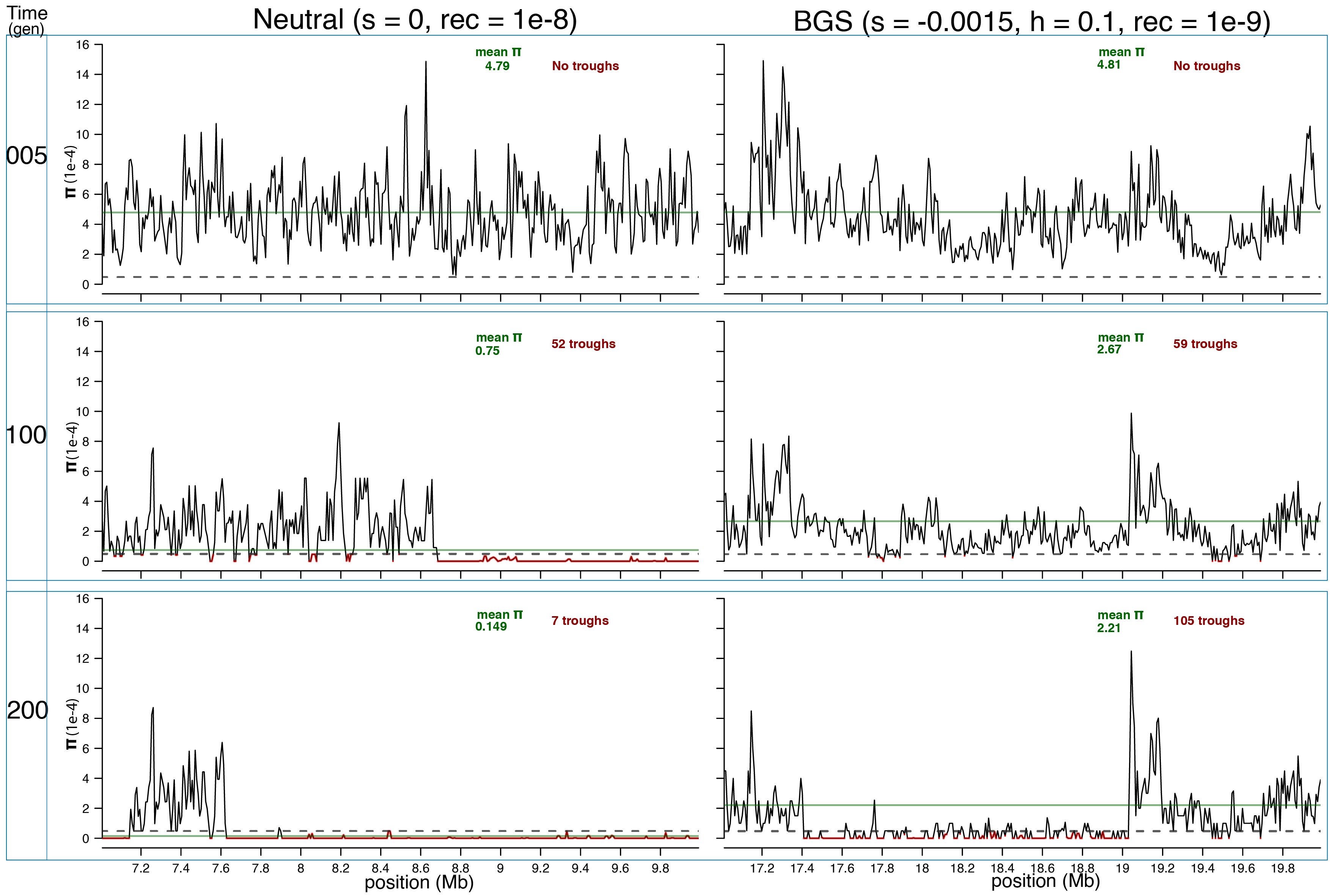


Figure S3: Genome scans of nucleotide diversity (π) during a bottleneck, for a neutral (left column) and a selected region (right column, h = 0.1). Three time points are shown: 5, 100 and 200 generations after the start of the bottleneck or a range expansion. Y-axis shows nucleotide diversity and X-axis shows position in the genome (only 3 Mb are shown here for clarity). Horizontal dotted line indicates through threshold (10% of average ancestral diversity within each region); horizontal solid green line shows the average diversity of the chromosome segment at the three-time point (and it is also annotated in green on the top of each panel). Troughs are highlighted in red, with their total number (whole genome) annotated in the top right in red. Note that in this specific comparison, the chromosome with partially recessive deleterious mutations has initially similar levels of diversity than the neutral segment, but it loses diversity less rapidly for a bottleneck of identical intensity.


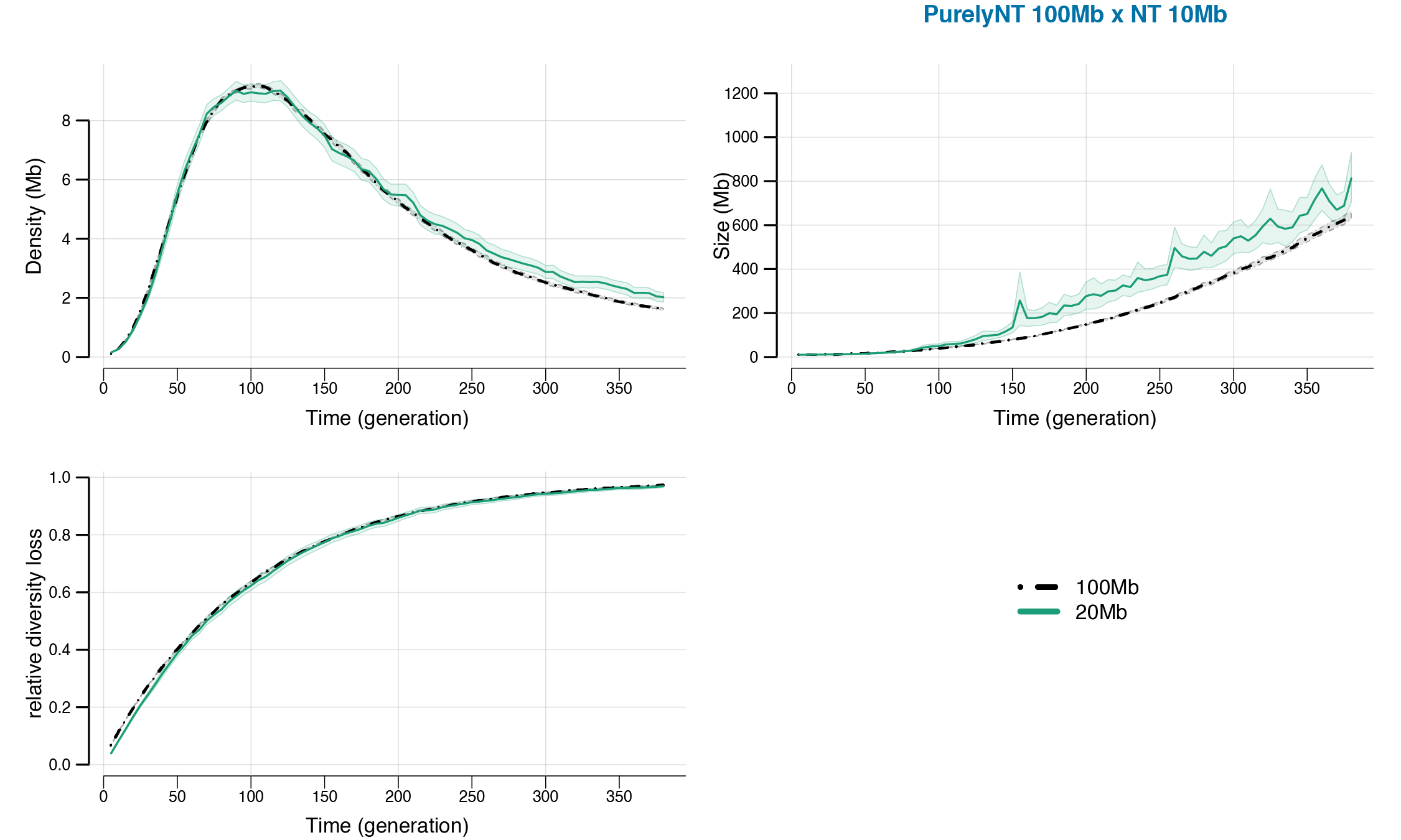


Figure S4: Effect of chromosome size on trough formation and genomic diversity during a bottleneck. All mutations are assumed here neutral. Trough density and relative diversity loss in chromosomes of 20 Mb are very similar to what is measured on 100Mb chromosomes. However, trough size is overestimated after 100 generations of bottleneck in chromosomes of 20 Mb. Shaded areas show 95% CI obtained from 10,000 bootstrap iterations.


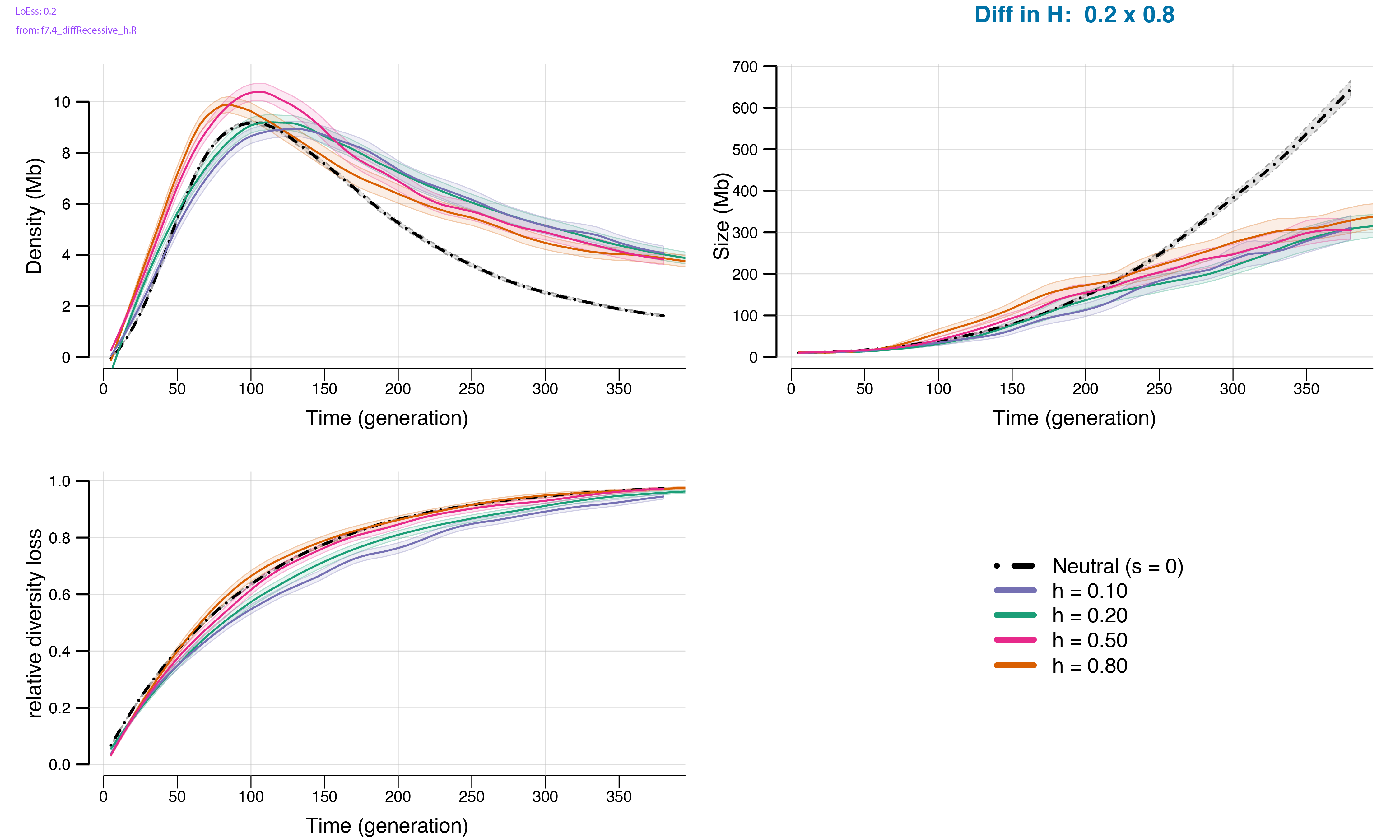


Figure S5: Properties of trough formation and genomic diversity loss during a bottleneck for chromosomes harboring mutation with difference dominance levels *h*. We show here simulations done with an intermediate recombination rate of 5 × 10^-9^ per bp per generation. Shaded areas show 95% CI obtained from 10,000 bootstrap iterations. The results were smoothed through a local polynomial regression (span = 0.2), see Material and Methods for details.


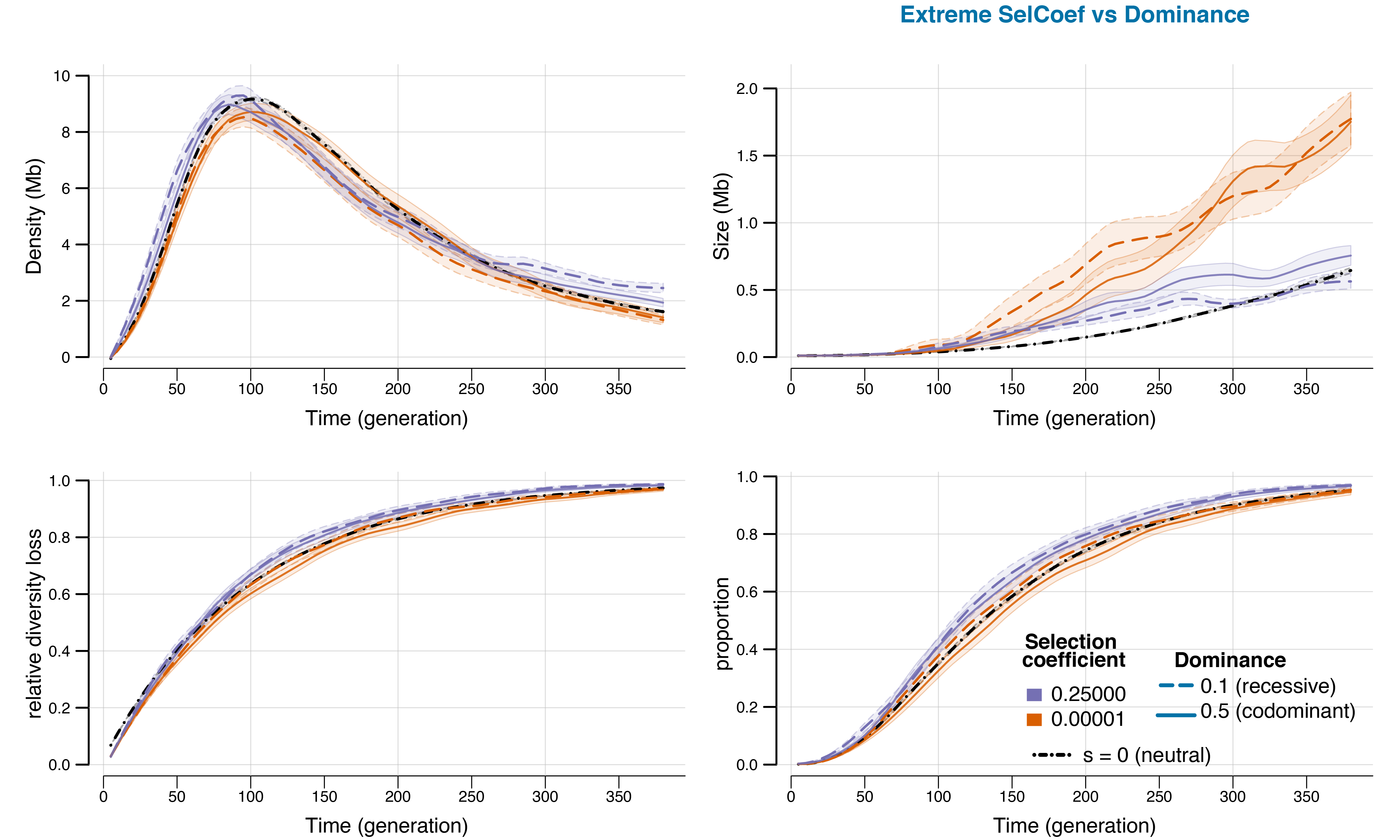


Figure S6: Trough statistics (density (A), size (B), relative diversity loss (C) and proportion of the genome within troughs (D)) for extreme selection coefficients (very weak s = 0.00001 and very strong s = 0.25). With these extremes values, trough formation and diversity loss evolve similarly to the neutral case, in line with the expectations that the effects of BGS are more prominent with intermediate selection coefficients (Nordborg et al. 1996; Good et al. 2014; Barroso and Ragsdale 2025). Shaded areas show 95% CI obtained from 10,000 bootstrap iterations. The results were smoothed through a local polynomial regression (span = 0.4), see Material and Methods for details.


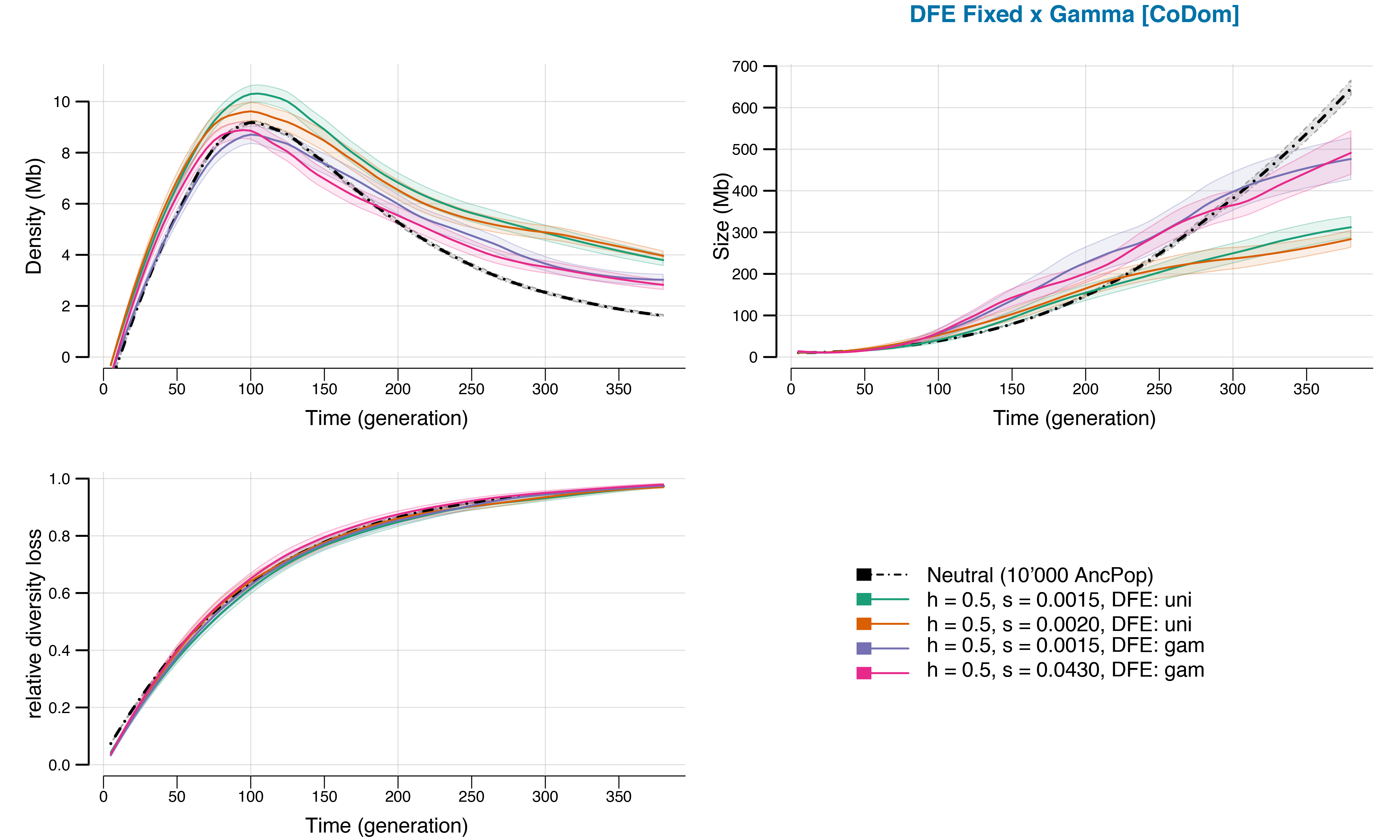


Figure S7: Properties of trough formation and genomic diversity loss during a bottleneck for chromosomes harboring codominant mutations (*h* = 0.5) and different distributions of selection coefficients, either of constant value (uni) or Gamma (gam) distributed values around a mean s equal to 0.0015 (purple) or 0.0430 (pink), both with shape parameter *a* = 0.23. Shaded areas show 95% CI obtained from 10,000 bootstrap iterations. The results were smoothed through a local polynomial regression (span = 0.4), see Material and Methods for details.


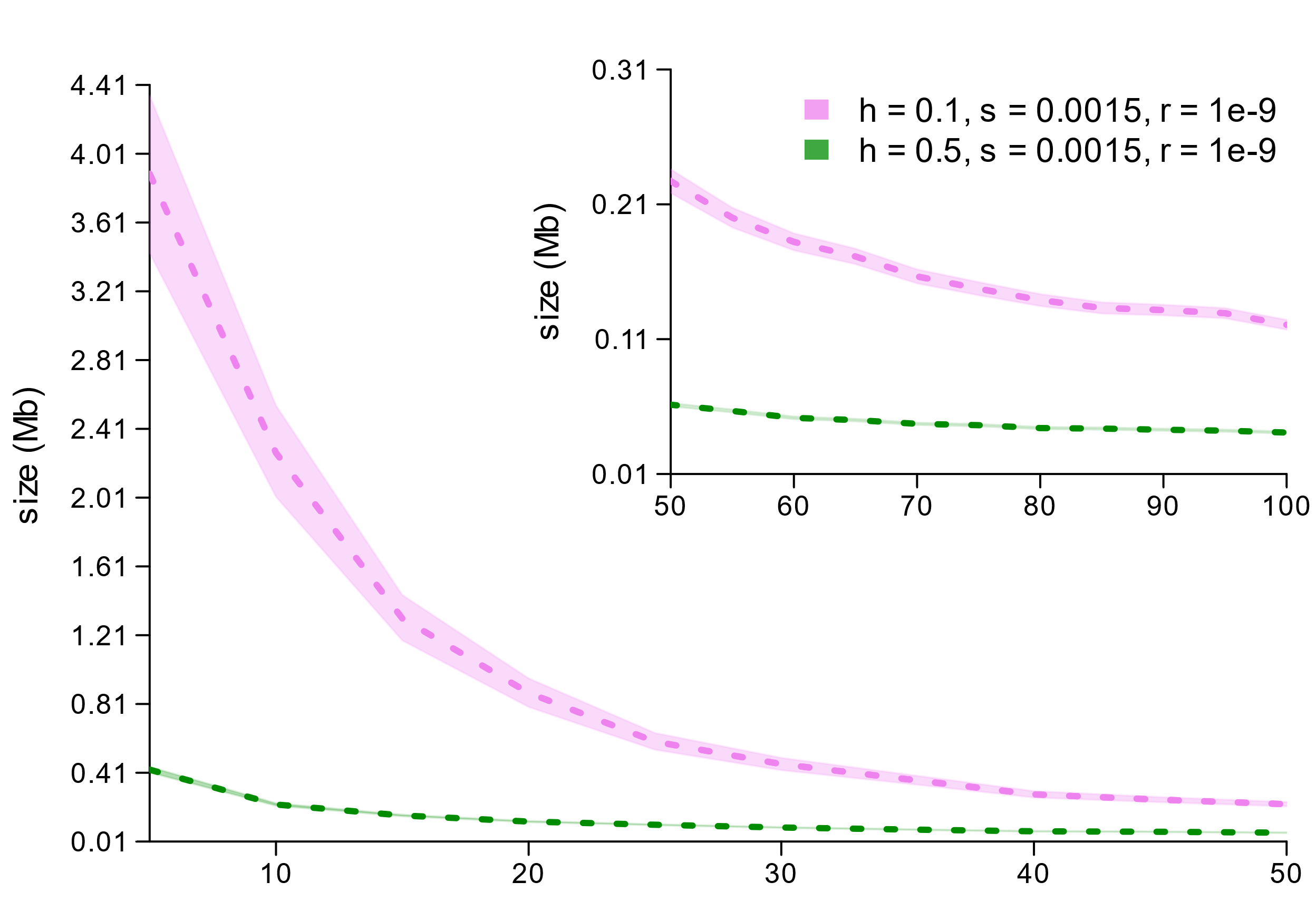


Figure S8: Size of diversity islands through time. There is a big difference between having codominant (green) and a recessive (pink) variants in terms of island sizes, and right at the beginning of the bottleneck, codominant islands are 400Kb long compared to ~4 Mb islands formed with highly recessive mutations. There is a quick decrease in island size in both cases, but the recessive mutations still maintain larger islands compared to the codominant variants after 100 generations (~120Kb versus ~40Kb). Shaded areas show 95% CI obtained from 10,000 bootstrap iterations.


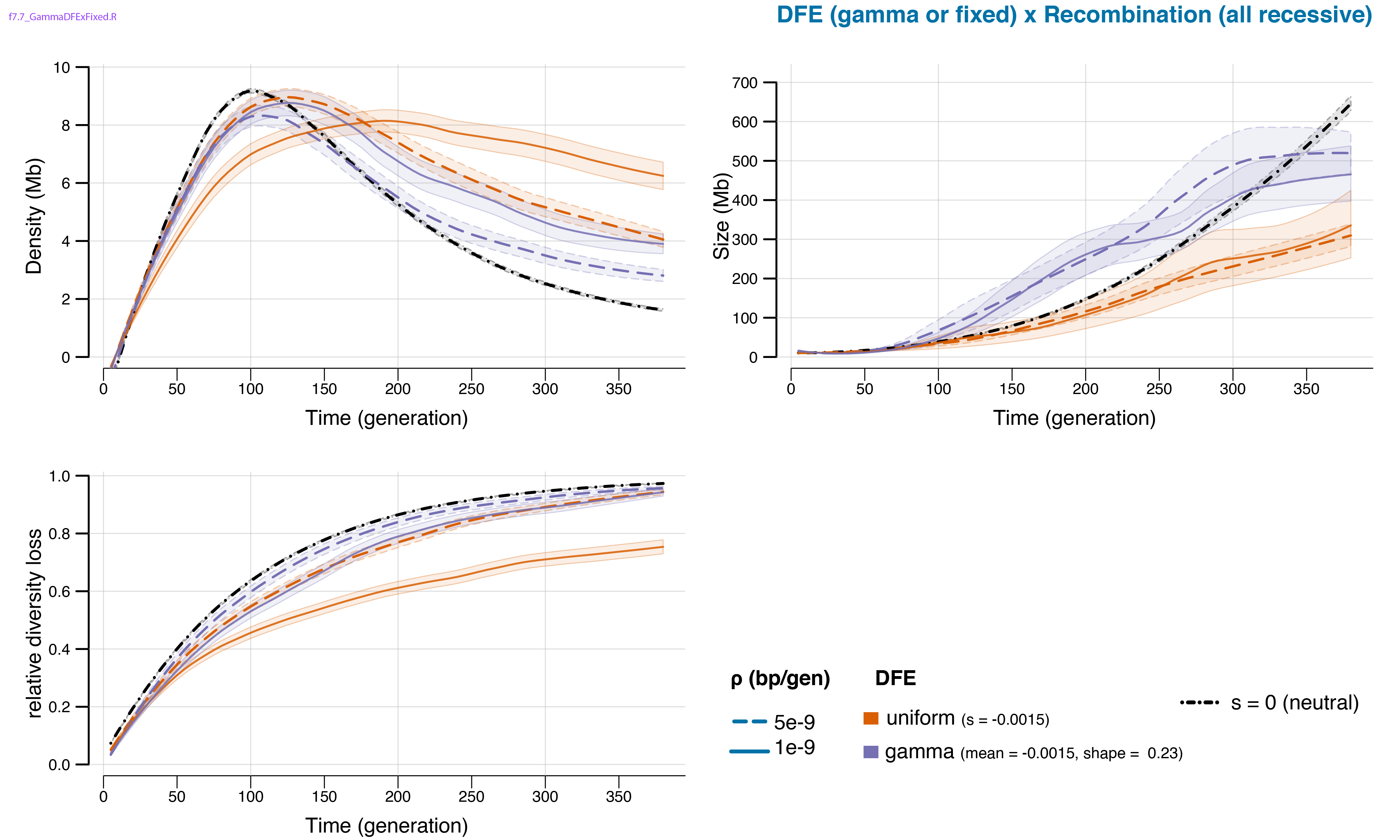
Figure S9: Properties of trough formation and genomic diversity loss during a bottleneck for chromosomes harboring highly recessive mutations (*h*=0.1) and different distributions of selection coefficients, either of constant value (s = 0.0015), or gamma distributed values (mean s = 0.0015, shape parameter = 0.23). The delay in diversity loss due to the non-random association of recessive variants still happens when a gamma DFE is used, albeit to a lesser extent. This is congruent with all previous results, considering that most mutations drawn from this gamma DFE will be nearly neutral. Shaded areas show 95% CI obtained from 10,000 bootstrap iterations. The results were smoothed through a local polynomial regression (span = 0.4), see Material and Methods for details.

**References**

Barroso GV, Ragsdale AP. 2025. A model for background selection in non-equilibrium populations. *bioRxiv* [Internet]:2025.02.19.639084. Available from: https://www.biorxiv.org/content/10.1101/2025.02.19.639084v1.abstract

Good BH, Walczak AM, Neher RA, Desai MM. 2014. Genetic Diversity in the Interference Selection Limit. *PLoS Genet.* 10:e1004222.

Nordborg M, Charlesworth B, Charlesworth D. 1996. The effect of recombination on background selection. *Genet. Res. (Camb.)* 67:159–174.

Schlichta F, Peischl S, Excoffier L. 2022. The Impact of Genetic Surfing on Neutral Genomic Diversity. *Mol. Biol. Evol.* 39:msac249.
